# Supplementary material for: Identification of Yak’s TLR4 Alternative Spliceosomes and Bioinformatic Analysis of TLR4 Protein Structure and Function
Source: Animals (Basel). 2020 Dec 26;11(1):32. doi: 10.3390/ani11010032 (PMC7823342; doi:10.3390/ani11010032)
Supplement: Supplementary file 1 [file animals-11-00032-s001.pdf]

animals

MDPI

Supplementary Materials

Identification of Yak's *TLR4* Alternative Spliceosomes and Bio-informatic Analysis of TLR4 Protein Structure and Function

Xingdong Wang, Jie Pei, Pengjia Bao, Chunnian Liang, Min Chu, Shaoke Guo, Ping Yan and Xian Guo

Table S1. RNA extraction metrics and tissue specific metadata.

Tissues	Amount Used by the Tissue (g)	RNA Concentration (Before Dilution) (ng/μL)	RNA OD260/280	RNA Concentration (After Dilution) (ng/μL)
Heart 1	3.07	650.8	1.92	500
Heart 2	2.94	648.2	1.99	500
Heart 3	3.12	701.3	2.04	500
Liver 1	2.78	883.1	1.96	500
Liver 2	2.92	941.6	1.96	500
Liver 3	3.05	1003.5	1.95	500
Lung 1	2.99	2100.6	2.06	500
Lung 2	2.86	2349.7	2.01	500
Lung 3	2.90	1977.5	2.00	500
Spleen 1	3.22	1922.4	1.99	500
Spleen 2	3.08	1500.7	1.93	500
Spleen 3	3.03	1673.0	2.04	500
Kidney 1	2.79	836.4	2.04	500
Kidney 2	2.86	1622.7	1.98	500
Kidney 3	3.01	1933.4	2.02	500
Muscle 1	3.11	583.7	1.97	500
Muscle 2	2.90	946.7	2.02	500
Muscle 3	2.83	762.1	1.92	500
Fat 1	2.93	600.4	1.96	500
Fat 2	2,80	716.2	2.07	500
Fat 3	3.01	1102.4	1.99	500
